# Surgeons’ opinions and concerns regarding prophylactic mesh placement when conducting a permanent ileo- and colostomy A survey among 172 surgeons in Germany, Switzerland, and Austria

**DOI:** 10.3389/fsurg.2024.1479870

**Published:** 2024-11-29

**Authors:** Christoph Paasch, Egan Leonidovich Kalmykov, Ralph Lorenz, Nele Neveling, Rene Mantke

**Affiliations:** ^1^ Department of Surgery, Brandenburg Medical School, University Hospital Brandenburg/Havel, Brandenburg, Germany; ^2^ Department of Vascular Surgery, Brandenburg Medical School, University Hospital Brandenburg/Havel, Brandenburg, Germany; ^3^ Hernia Center, Berlin, Germany; ^4^ Faculty of Health Science Brandenburg, Brandenburg Medical School, University Hospital Brandenburg/Havel, Brandenburg, Germany

**Keywords:** parastomal hernia, guideline, non-absorbable mesh, prophylactic mesh placement, hernia

## Abstract

**Background:**

Prophylactic mesh placement when creating a permanent colostomy was recommended by the 2017 European Hernia Society guidelines on the prevention and treatment of parastomal hernias (GPTPH2017). The extent of this recommendation is under debate based on the long-term data from clinical trials. Our aim was to conduct a survey of surgeons revealing perspectives and concerns regarding GPTPH2017 and to discuss their concerns.

**Methods:**

From January 2023 to September 2023 a survey among surgeons of Germany, Switzerland and Austria was conducted. The questionnaire addressed demographic data of the participants, information on work experience/location, number of elective permanent colo- and ileostomies, and opinions on the recommendation of GPTPH2017 for prophylactic mesh placement.

**Results:**

A total of 172 surgeons from Germany, Austria and Switzerland answered the questionnaire and 59 of them stated professional experience of 20–30 years. Most of the surgeons (*n* = 51, 31.3%) worked in a primary care hospital. A total of 112 participants were familiar with the GPTPH2017. Sixty-five surgeons (40%) stated that they never conduct a prophylactic mesh placement when creating an elective permanent colostomy (rarely, *n* = 44 (26.7%). Seven participants always place a mesh (4.2%, missing data: 7). Main concerns regarding prophylactic mesh placement was the concern of surgeons about wound infection (*n* = 107, 67.7%) and lack of evidence (*n* = 65, 41.1%). For some participants the GPTPH2017 is seen to be industry-driven with low evidence, too old and leading to overtreatment.

**Conclusions:**

The main reason for not placing a prophylactic mesh when conducting a permanent colostomy was the risk of wound infection.

## Introduction

The sufficient treatment and prevention of a parastomal hernia is important because this health condition frequently occurs when creating an end colostomy, with the incidence occurring up to 64% (1–3). In order to reduce these incidence, prophylactic mesh placement (PMP) has been studied in several randomized clinical trials (4, 5). A reduced rate of parastomal hernias was detected in the majority of the studies (4–6) and based on these findings, the European Hernia Society created a guideline in 2017 (7).

From our experience the guideline has not been implemented into daily routine, and therefore we conducted a German nationwide hospital discharge data analysis (*n* = 41,697) in 2023. We focused on prophylactic PMP after rectal resection without sphincter preservation. The rate of PMP only increased from 0.2% (*n* = 8) in 2010 to 6.4% (*n* = 198) in 2020 (8). This indicated an insufficient guideline implementation.

One reason could be related to current published (after 2017) literature with long-term results (≥5 years, *n* = 194 from 3 randomized clinical trials). In these studies, the PMP did not, with significance, prevent parastomal hernias after creating a permanent colostomy (9–12). Therefore, an updated guideline was published in September 2023 (12, 13). The recommendation changed from *strong* to *conditional recommendation for the use of a prophylactic mesh in patients with an end colostomy and fair life expectancy*. A strong recommendation for the use of a prophylactic mesh in patients at high risk to develop a parastomal hernia was still stated (12, 13).

Beyond these published long-term results and guideline changes, we wanted to obtain more information about surgeons’ opinions and concerns about PMP in the placement of a permanent colostomy to gain insight into the reality of care.

## Methods

From January 2023 to September 2023 a survey among surgeons of Germany, Switzerland and Austria was conducted at the University hospital Brandenburg an der Havel. The survey was approved by the Brandenburg Medical School Theodor Fontane on the 11th of August in 2023 (E-01-20230710) and was conducted in accordance with the ethical standards of the Helsinki Declaration 1975.

A comprehensive online questionnaire, constituted of 13 PH-focused queries, was used (UmfrageOnline©2007–2023 enuvo GmbH; www.umfrageonline.com). The graphs and figures were generated by that software and the results checked by the first author. “The participants gave their consent to participate in the survey.”

### Study design

The questionnaires were sent out two times by three investigators to hospitals in Germany, Austria and Switzerland.

The university hospitals in Germany and those hospitals of the HELIOS and SANA were contacted. In terms of Austria and Switzerland, the investigator performed an online search for hospitals with surgical departments.

The main objective was to collect data from different types of hospitals. Therefore, all university hospitals were contacted. It was requested that the survey be forwarded to the employees. HELIOS and SANA were selected because they are the largest hospital associations in Germany. They include all types of hospitals. In addition, their websites provide a sufficient overview of the contact details of each hospital. As we wanted a perspective of other countries randomly hospitals in Austria (*n* = 14) and Switzerland (*n* = 2) were contacted.

The questionnaire consisted of 13 queries:

1. How old are you?
2. What sex do you have?
3. How many years have you worked in general and visceral surgery?
4. In which institution are you clinically active?
5. How many permanent ileostomies are performed in your clinic per year?
6. How many permanent colostomies are performed in your clinic per year?
7. In which state/province is your place of work located?
8. Are you familiar with the guidelines for the prevention and treatment of parastomal hernias published in 2017 (GPTPH2017)?
9. How did you become aware of the guidelines? (Multiple answers possible)
10. How often do you implant a mesh in your clinic after elective creation of a permanent colostomy?
11. How often do you implant a mesh in your clinic after elective creation of a permanent ileostomy? Multiple answers possible
12. From your point of view, what aspects speak against a preventive mesh placement?
13. Which aspects of the guideline do they see critically? Multiple answers possible.

### Primary objective

The primary objective was to identify the reasons why surgeons do not conduct prophylactic mesh placement.

### Secondary objectives

The secondary objective was to determine what aspects of GPTPH2017 are seen critically and the number of prophylactic mesh placements in Germany, Switzerland, and Austria.

### Statistical analysis

A descriptive data analysis was performed using Microsoft excel. No exploratory analyses took place.

## Results

### Baseline characteristics of participants

At the time of survey conduction 172 surgeons answered the questionnaire, of which 30 were women and 134 men (Missing data: 8). The average age was 48.12 years (standard deviation: 9.99; missing data: 38).

A total of 59 participants reported professional experience of 20–30 years and 42 individuals have been practicing as surgeons for 11–20 years. A total of 32 participants have professional experience of more than 30 years and 21 have been working as surgeons for 6–10 years. The minority of 10 participants have work experience of 0–5 years (Missing data: 8).

Most participants (*n* = 51, 31.29%) worked in a primary care hospital (According to WHO: primary care). A total of 43 (26.38%) surgeons were employed in a hospital for specialized care (According to WHO: secondary care) and 38 (23.31%) surgeons worked in a maximum care hospital (According to WHO: tertiary care). In 29 (17.79%) cases the participants were employed in a university hospital (According to WHO: tertiary care) and two worked (1.23%) in the outpatient sector (According to WHO: primary care; Missing data: 9).

**Figure 1:**
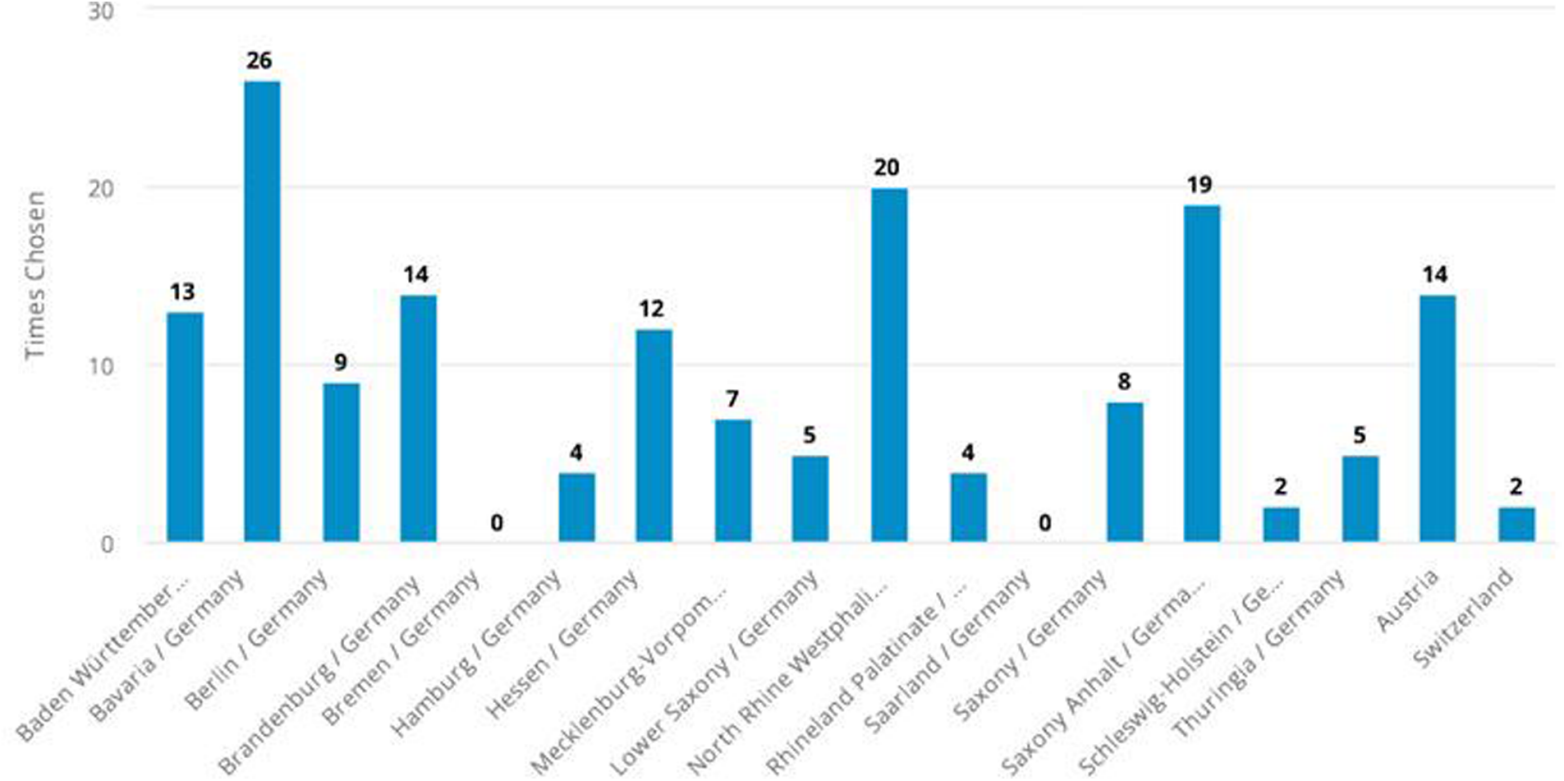
Information about the participants’ workplace (missing data: 7).

The countries/states of the participants are depicted in Figure 1.

### Information's regarding the prevention and treatment of parastomal hernias

A total of 112 (68.29%) participants were familiar and 52 (31.71%) were not familiar with the GPTPH2017 (Missing data: 8).

Among those who were familiar with this recommendation, 79 surgeons stated that they become aware of them during meetings and congresses, 29 participants were informed by fellow workers and 29 individuals by an announcement of their professional society. A total of 14 surgeons stated they received the information from social media and press.

A total of 66 (40.42%) individuals stated that they conducted between 0 and 5 elective permanent ileostomies per year. A total of 68 (41.46%) surgeons reported that they performed between 10 and 20 elective permanent colostomies per year. Information on the annual number of times an elective permanent ileostomy and colostomy is performed is shown in Supplementary Figures S1 and S2.

A total of 99 participants (60%) stated that they never conduct a prophylactic mesh placement, when creating a permanent ileostomy (rarely, *n* = 42 (25.45%; occasionally, *n* = 15 (9.09%); often, *n* = 6 (3.64%); always, *n* = 3 (1.82%); missing data: 7; Supplementary Figure S3).

A total of 65 participants (39.99%) stated that they never conduct a prophylactic mesh placement, when creating a permanent colostomy (rarely, *n* = 44 (26.67%); occasionally, *n* = 32 (19.39%); often, *n* = 17 (10.30%); always, *n* = 7 (4.24%); missing data: 7; Figure 2).

**Figure 2:**
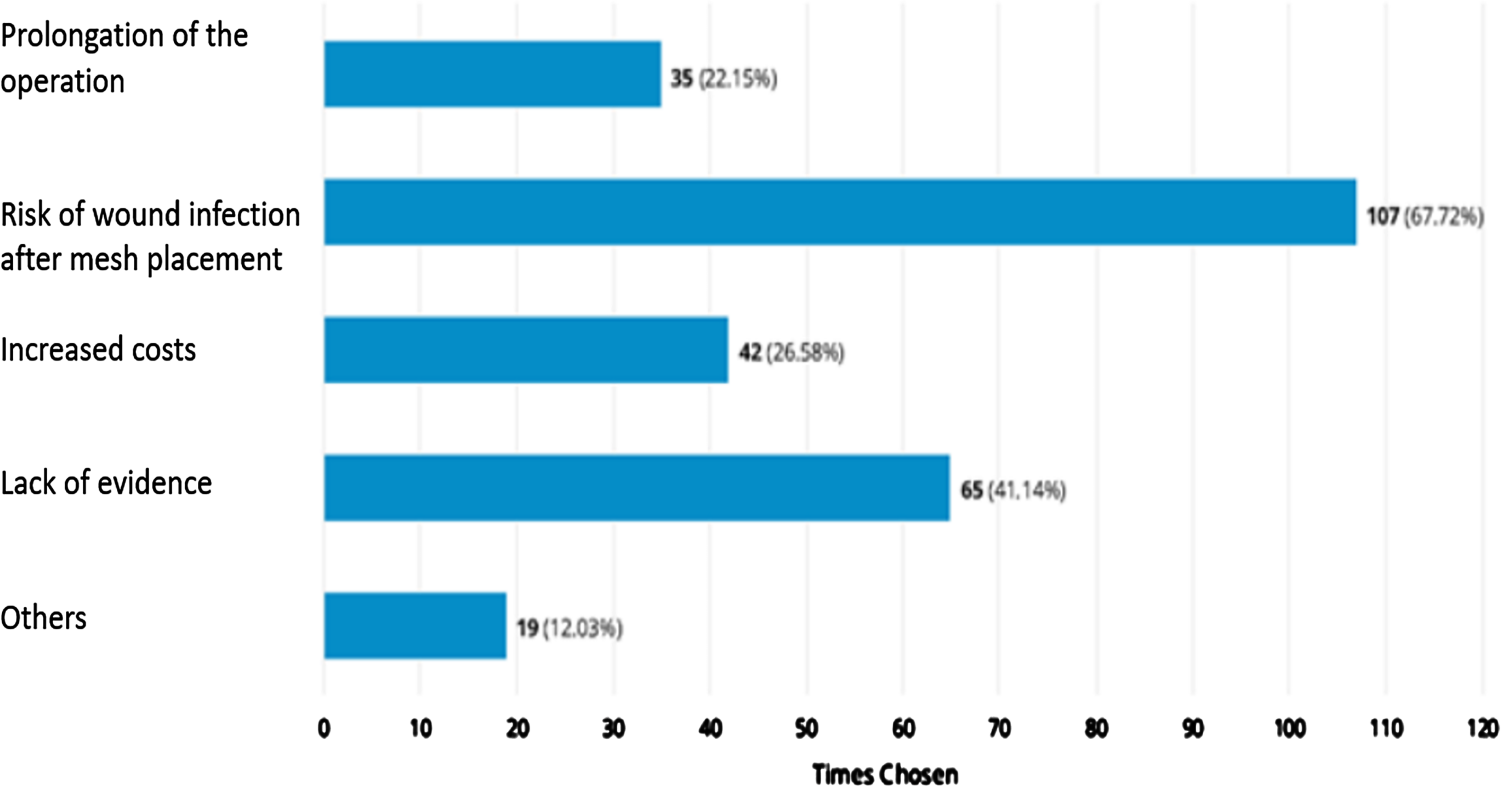
Prophylactic mesh placement when creating a permanent colostomy. Missing data: 7.

### Perspectives and concerns regarding prophylactic mesh placement and the GPTPH2017

**Figure 3:**
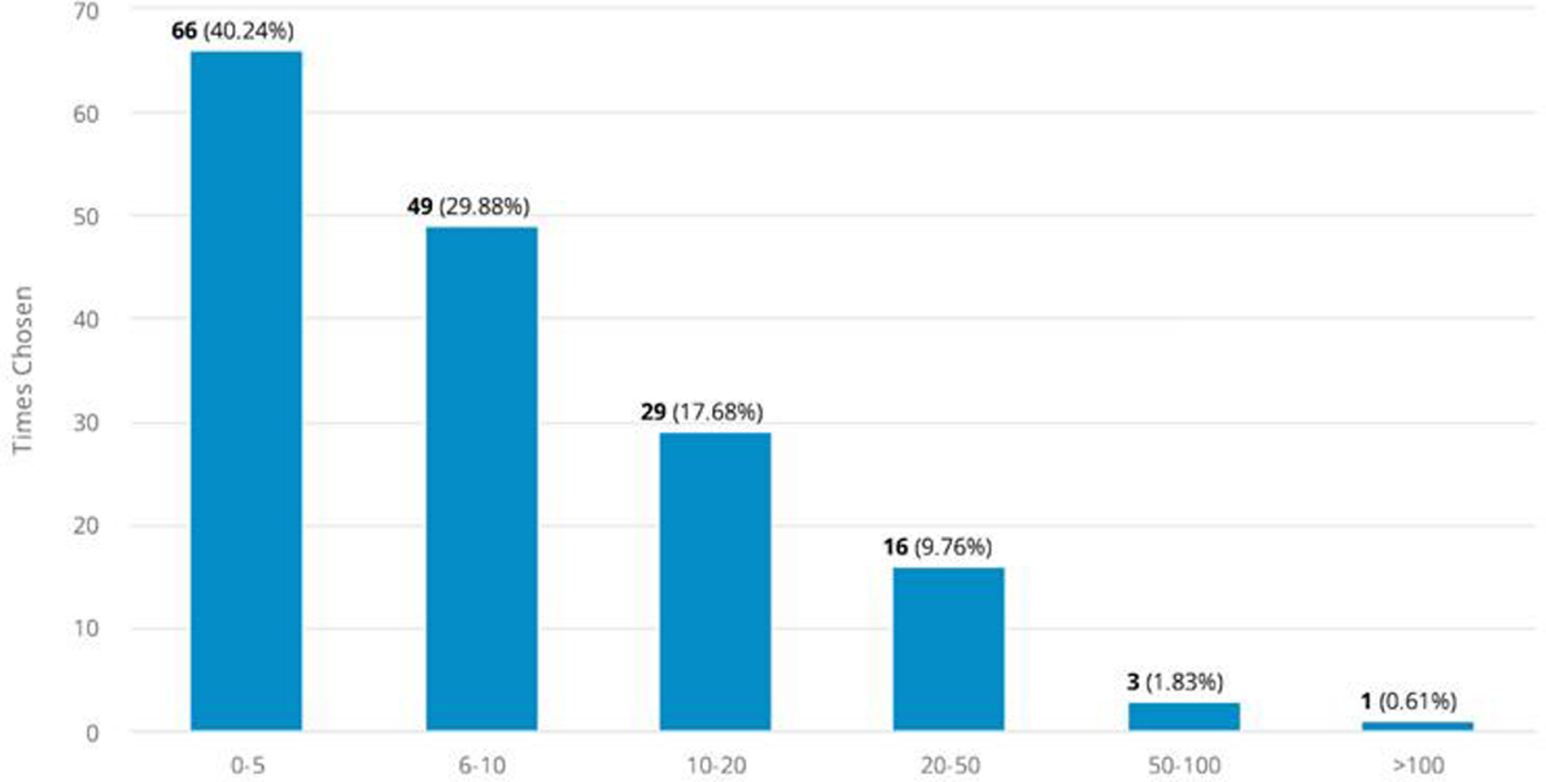
Perspectives and concerns on prophylactic mesh placement and parastomal guidelines (missing data: 14).

The aspects that, in the opinion of the respondents, speak against a preventive mesh placement are shown in Figure 3. Other reasons were in detail:

• “Complication makes revision more difficult”
• “We only create stomas in the context of emergency interventions, mostly in the case of peritonitis, and then we do not use any foreign material.”
• “Mesh related complication”, “none” (*n* = 7)
• “Extremely difficult revision conditions e.g., with stoma stenosis”
• “bowl erosions”
• “High number needed to treat. Many get a mesh unnecessarily.”
• “Different technique, lateralisation, Dynamesh with “chimney” - without lateralization keyhole on the net?”
• “Approx. 50% of patients are overtreated with a mesh.”
• “Bowel lesion due to a mesh”
• “In our clinic, we have had very bad experience with mesh implantation in ostomies. Mesh migration and revision.”
• “No mesh implantation for ileostomy or ilumconduit.”
• “I have treated many patients with symptomatic parastomal hernia for ilumconduit also treated with mesh.”

The following aspects were considered critical in terms of GPTPH2017:

• “Basically none, a guideline is a guideline, not an absolute must, i.e., there is always some leeway”
• “mesh placement”
• “According to Schrempf and Anthuber 2019, no advantages of mesh implantation, elect. two-stage lap. treatment with IPOM standard in our clinic if clinically relevant hernia formation”
• “Lack of evidence” (*n* = 7)
• “industry-triggered”
• “Guideline is >7 years old!”
• “No national guideline”
• “The Swedish study shows no difference in the rate of parastomal hernias with prophylactic mesh implantation”.
• “I treat parastomal hernias with a Dahlhausen 3-D mesh. This works excellently. I would also place these prophylactically, but they are simply too expensive for that. A normal net is insufficient.”
• “The special IPST mesh is very good for secondary placement. Not all patients develop or experience significant herniation. I am therefore critical of the prophylactic insertion.”

No criticism was stated in two cases (Table 1).

**Table 1:** Summarized concerns regarding GPTPH2017.

- Lack of evidence
- Guideline is too old
- Guideline is industry-triggered
- New evidence partial contradicting prophylactic mesh placement

European Hernia Society guidelines on the prevention and treatment of parastomal hernias 2017 GPTPH2017.

## Discussion

PMP when creating a permanent colostomy was recommended by the 2017 European Hernia Society guidelines on the prevention and treatment of parastomal hernias (GPTPH2017). This recommendation was limited to just three randomized clinical trials and the long-term follow-up data were published and summarized by Lopez-Cano et al. (2022). However, the guidelines on the treatment of parastomal hernias have been revisited in two publications from 2023 (12, 13).

A total of 65 survey participants in our study stated that the lack of evidence speaks against PMP. Some participants mentioned the “Swedish study” and the authors Schrempf and Anthuber (14). They published a journal club paper discussing the Swedish publication by Odensten et al. (2019). This research group published the results of the randomized controlled double-blinded multicenter STOMAMESH trial (*n* = 211, PMP vs. No-PMP) (15). After 1 year no differences in terms of the rate of parastomal hernias were revealed and these findings seem to be in accordance with published long-term results by López-Cano et al. (2022). Lopez-Cano et al. (2022), analyzed three randomized clinical trials (*n* = 121; median overall length of 48.5 months) and found that 82 (67.8%) individuals developed a parastomal hernia, diagnosed by computed tomography (Mesh group, *n* = 30 vs. no PMP, *n* = 52). PMP did not significantly lower the hernia rate (2, 9, 10, 16). However, in the Lopez-Cano et al. (2022) study, on the other hand, the amount of individuals analyzed was low and the dropout rate ranged from 32%–50% (mostly due to death within 5 years after surgery). In all three trials a trend towards a lower rate of parastomal hernia when a PMP took place was observed. Furthermore, in a publication by Jänes et al. (2009), that had a 5-year follow-up, PMP reduced the rate of parastomal hernia, with significance (No-PMP 17/21 (81%) vs. 2/15 (13.3%) (10). Based on these studies it seems reasonable to conditionally recommend PMP (12, 13). However, other research shows that PMP did not significantly lower the hernia rate (2, 9, 10, 16). The survey we conducted shows that the participants are aware of this ongoing debate and the weakened evidence on this topic most likely led to the insufficient guideline implementation we reported before (8).

Participants stated, “High number needed to treat”, “Approx. 50% of patients are overtreated with a mesh”, and “Not all patients develop or experience significant herniation” in our survey. This aspect is still under debate, as it is related to long-term recurrence rates. The low rate of recurrences reported by Jänes et al. (2009), when PMP took place and the high rate without would not support the statement on overtreatment (10).

Survey participants also viewed the PMP recommendation as “industry-driven”. There is an ongoing discussion on advantages and disadvantages of industry-funded research and we did review the guideline literature on PMP in terms of funding and author disclosures. We identified 13 relevant randomized clinical trials and a total of 6 were published after GPTPH2017. Two trials were funded by the industry and a PMP was not recommended by these authors (9, 17). Four trials did not report information on funding to the best of our knowledge, and in 9 of the 13 trials the authors did not have any disclosures (4–6, 9, 11, 15, 17–19). From our perspective, after reviewing literature, PMP recommendations being “industry-driven” seems to be an overstatement. Nevertheless, in some cases there is a lack of transparency and transparency is essential.

A total of 107 participants stated that a risk of wound infection speaks against prophylactic mesh placement when creating a permanent end colostomy. But there are low rates of wound infections publish in the literature as seen by DeAsis et al. (2015). DeAsis et al. (2015), reported a 3.8% wound infection rate when meta-analysing 469 cases of laparoscopic parastomal hernia repair (20). Higher rates were revealed by a research group from Finland, where the authors retrospectively analyzed 235 individuals, who underwent elective parastomal hernia repair (Sugarbaker (38.8%), keyhole (16.3%), sandwich techniques (15.4%), other (29.5%)). The median follow-up time was 39.0 months, only two fistulas and two mesh removals were reported, and the wound infection rate was 8.9% (21). In terms of PMP two randomized clinical trials with a long-term follow-up stated no mesh erosions, fistulas, and wound infection (10, 11).

Some participants stated that the guideline is too old and it is not mandatory to follow them: “Basically none, a guideline is a guideline, not an absolute must, i.e., there is always some leeway”. The publication of an updated version of the guideline was overdue and has fortunately been done (12, 13). The survey results show that surgeons from Germany, Switzerland and Austria did not follow these guidelines strictly and that current literature appears to be continually reviewed. A total of 112 (68.29%) participants were familiar with the GPTPH2017. Sixty-five surgeons (39.99%) stated that they never conduct a prophylactic mesh placement, when creating an elective permanent colostomy.

The topic is also of interest from a sustainability perspective. This is because the medical sector is responsible for up to 5% of annually greenhouse gas emissions worldwide, In surgery, emissions are assumed to be 6 times higher than in other areas of the hospital (22). If the PMP would lead to less future incisional repairs emissions could actually be reduced on a large scale.

### Limitation

In two cases the participants worked in the outpatient sector, and this survey was not intended to reach surgeons outside of a hospital setting. We assume that the link was forwarded by a surgeon working in a hospital. Another limitation is the lack of more detailed information on participants’ experiences of undergoing PMP, ileostomy and colostomy.

The limited external validity is a significant concern, as the results are based on a sample of surgeons from German-speaking countries only. This could restrict the generalizability of the findings to other international contexts. Moreover, acknowledging the lack of detailed information on the participants’ experiences with prophylactic mesh could have limited the depth of the responses collected.”

## Conclusion

Most participants were familiar with the GPTPH2017 and about 40% never conducted a prophylactic mesh placement when creating an elective permanent colostomy. The main reason for not placing a prophylactic mesh when conducting a permanent colostomy was the concerns of surgeons about wound infections.

## Data Availability

The original contributions presented in the study are included in the article/Supplementary Material, further inquiries can be directed to the corresponding author/s.

## Supplementary material

Supplementary Figure S1

Annual number of times an elective permanent ileostomy is performed. Missing data: 7.

Supplementary Figure S2

Annual number of times an elective permanent colostomy is performed. Missing data: 7.

Supplementary Figure S3

Prophylactic mesh placement when creating a permanent ileostomy. Missing data: 7.

## References

[1] Liu L Zheng L Zhang M Hu J Lu Y Wang D. Incidence and risk factors for parastomal hernia with a permanent colostomy. J Surg Oncol. (2022) 126(3):535–43. 10.1002/jso.26919 35608292

[2] Makarainen E Rautio T Rintala J Muysoms F Kauppila JH. Incidence of parastomal and incisional hernia following emergency surgery for Hinchey III-IV diverticulitis: a systematic review. Scand J Surg. (2022) 111(2):14574969221107276. 10.1177/14574969221107276 35748305

[3] Lopez-Cano M Serra-Aracil X Mora L Sanchez-Garcia JL Jimenez-Gomez LM Marti M Preventing parastomal hernia using a modified sugarbaker technique with composite mesh during laparoscopic abdominoperineal resection: a randomized controlled trial. Ann Surg. (2016) 264(6):923–8. 10.1097/SLA.0000000000001684 27828820

[4] Ringblom C Odensten C Strigard K Gunnarsson U Nasvall P. No reduction in parastomal hernia rate 3 years after stoma construction with prophylactic mesh: three-year follow-up results from STOMAMESH—a multicenter double-blind randomized controlled trial. Ann Surg. (2023) 277(1):38–42. 10.1097/SLA.0000000000005537 35837972 PMC9762699

[5] Marinez AC Bock D Erestam S Engstrom A Kalebo P Nielsen YW Methods of colostomy construction: no effect on parastomal hernia rate: results from stoma-const—a randomized controlled trial. Ann Surg. (2021) 273(4):640–7. 10.1097/SLA.0000000000003843 32209907

[6] Lambrecht JR Larsen SG Reiertsen O Vaktskjold A Julsrud L Flatmark K. Prophylactic mesh at end-colostomy construction reduces parastomal hernia rate: a randomized trial. Colorectal Dis. (2015) 17(10):O191–7. 10.1111/codi.13065 26179984

[7] Antoniou SA Agresta F Garcia Alamino JM Berger D Berrevoet F Brandsma HT European Hernia society guidelines on prevention and treatment of parastomal hernias. Hernia. (2018) 22(1):183–98. 10.1007/s10029-017-1697-5 29134456

[8] Paasch C Kobelt E Lunse S Heisler S Lorenz R Hunger R How often is prophylactic parastomal mesh placement performed after rectal resection without sphincter preservation? An analysis of German nationwide hospital discharge data among 41,697 patients. Hernia. (2023) 28(1):9–15. 10.1007/s10029-023-02887-9 37843603 PMC10891180

[9] Brandsma HT Hansson BM Aufenacker TJ de Jong N Engelenburg KCV Mahabier C Prophylactic mesh placement during formation of an end-colostomy: long-term randomized controlled trial on effectiveness and safety. Ann Surg. (2023) 278(3):e440–e6. 10.1097/SLA.0000000000005801 36727747

[10] Janes A Cengiz Y Israelsson LA. Preventing parastomal hernia with a prosthetic mesh: a 5-year follow-up of a randomized study. World J Surg. (2009) 33(1):118–21; discussion 22-3. 10.1007/s00268-008-9785-4 19011935

[11] Makarainen-Uhlback EJ Klintrup KHB Vierimaa MT Carpelan-Holmstrom MA Kossi JAO Kairaluoma MV Prospective, randomized study on the use of prosthetic mesh to prevent a parastomal hernia in a permanent colostomy: results of a long-term follow-up. Dis Colon Rectum. (2020) 63(5):678–84. 10.1097/DCR.0000000000001599 32032196

[12] Stabilini C Muysoms FE Tzanis AA Rossi L Koutsiouroumpa O Mavridis D EHS rapid guideline: evidence-informed European recommendations on parastomal hernia prevention—with ESCP and EAES participation. J Abdom Wall Surg. (2023) 2. 10.3389/jaws.2023.11549 38312414 PMC10831651

[13] Tzanis AA Stabilini C Muysoms FE Rossi L Koutsiouroumpa O Mavridis D Update systematic review, meta-analysis and GRADE assessment of the evidence on parastomal hernia prevention—a EHS, ESCP and EAES collaborative project. J Abdom Wall Surg. (2023) 2. 10.3389/jaws.2023.11550 38312423 PMC10831653

[14] Schrempf M Anthuber M. Prophylactic implantation of mesh does not prevent parastomal hernia after colostomy creation. Chirurg. (2019) 90(5):416. 10.1007/s00104-019-0933-1 30874864

[15] Odensten C Strigard K Rutegard J Dahlberg M Stahle U Gunnarsson U Use of prophylactic mesh when creating a colostomy does not prevent parastomal hernia: a randomized controlled trial-STOMAMESH. Ann Surg. (2019) 269(3):427–31. 10.1097/SLA.0000000000002542 29064900 PMC6369967

[16] Lopez-Cano M Adell-Trape M Verdaguer-Tremolosa M Rodrigues-Goncalves V Badia-Closa J Serra-Aracil X. Parastomal hernia prevention with permanent mesh in end colostomy: failure with late follow-up of cohorts in three randomized trials. Hernia. (2023) 27(3):657–64. 10.1007/s10029-023-02781-4 36966221 PMC10220116

[17] Fleshman JW Beck DE Hyman N Wexner SD Bauer J George V A prospective, multicenter, randomized, controlled study of non-cross-linked porcine acellular dermal matrix fascial sublay for parastomal reinforcement in patients undergoing surgery for permanent abdominal wall ostomies. Dis Colon Rectum. (2014) 57(5):623–31. 10.1097/DCR.0000000000000106 24819103

[18] Vierimaa M Klintrup K Biancari F Victorzon M Carpelan-Holmstrom M Kossi J Prospective, randomized study on the use of a prosthetic mesh for prevention of parastomal hernia of permanent colostomy. Dis Colon Rectum. (2015) 58(10):943–9. 10.1097/DCR.0000000000000443 26347966

[19] Tarcoveanu E Vasilescu A Cotea E Vlad N Palaghia M Danila N Parastomal hernias – clinical study of therapeutic strategies. Chirurgia (Bucur). (2014) 109(2):179–84. 24742407

[20] DeAsis FJ Lapin B Gitelis ME Ujiki MB. Current state of laparoscopic parastomal hernia repair: a meta-analysis. World J Gastroenterol. (2015) 21(28):8670–7. 10.3748/wjg.v21.i28.8670 26229409 PMC4515848

[21] Makarainen-Uhlback E Vironen J Falenius V Nordstrom P Valikoski A Kossi J Parastomal hernia: a retrospective nationwide cohort study comparing different techniques with long-term follow-up. World J Surg. (2021) 45(6):1742–9. 10.1007/s00268-021-05990-z 33560501 PMC8093171

[22] Rizan C Steinbach I Nicholson R Lillywhite R Reed M Bhutta MF. The carbon footprint of surgical operations: a systematic review. Ann Surg. (2020) 272:986–95. 10.1097/SLA.0000000000003951 32516230

