# Supplementary figures and images for: Surgeons’ opinions and concerns regarding prophylactic mesh placement when conducting a permanent ileo- and colostomy A survey among 172 surgeons in Germany, Switzerland, and Austria
Source: Front Surg. 2024 Nov 29;11:1479870. doi: 10.3389/fsurg.2024.1479870 (PMC11638169; doi:10.3389/fsurg.2024.1479870)

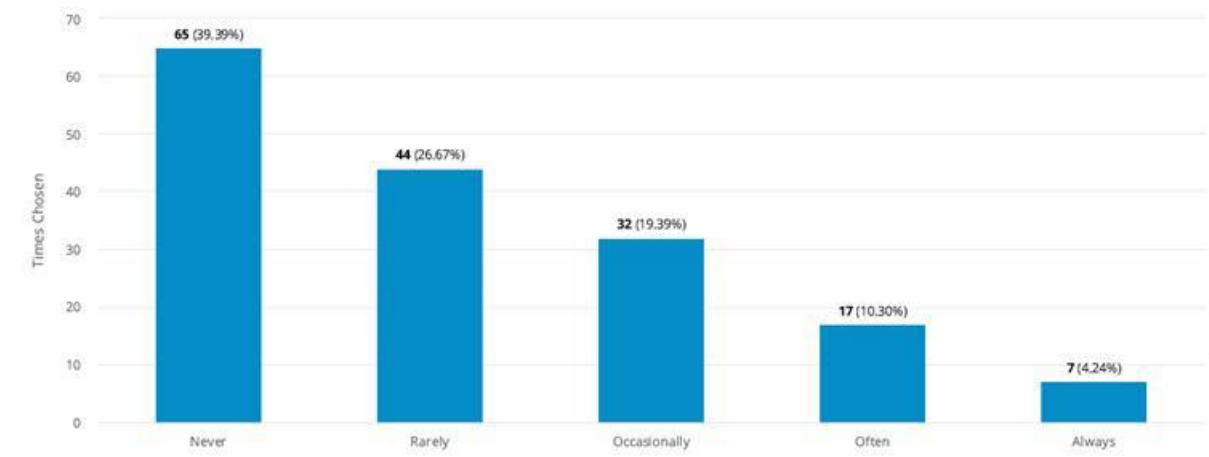

S1

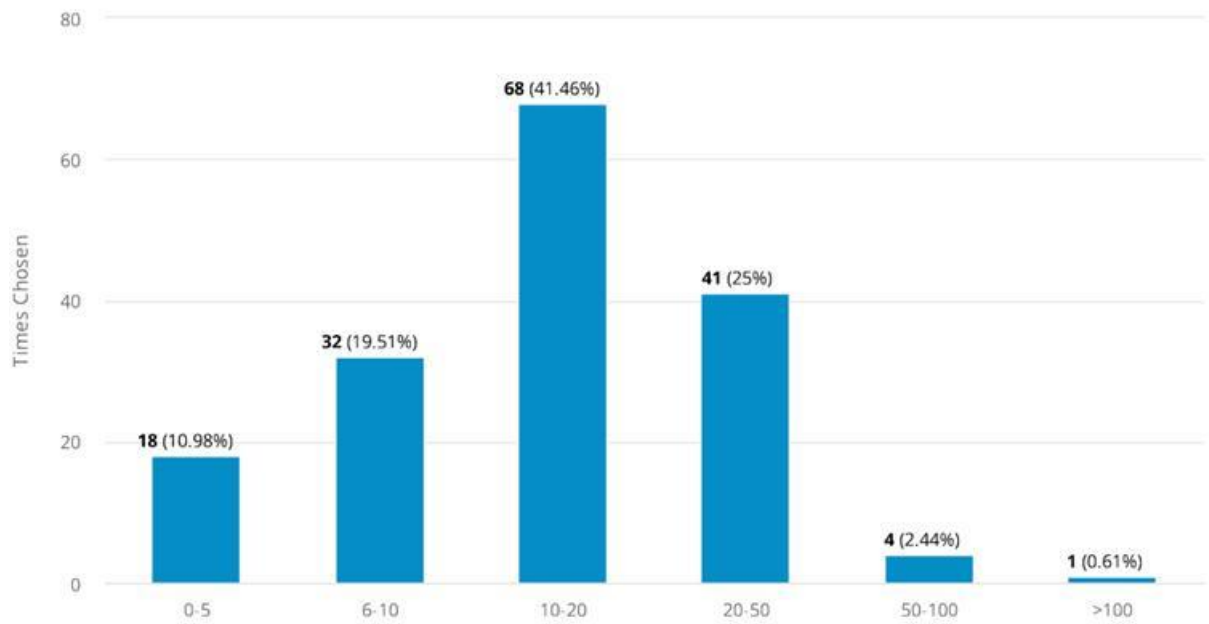

S2

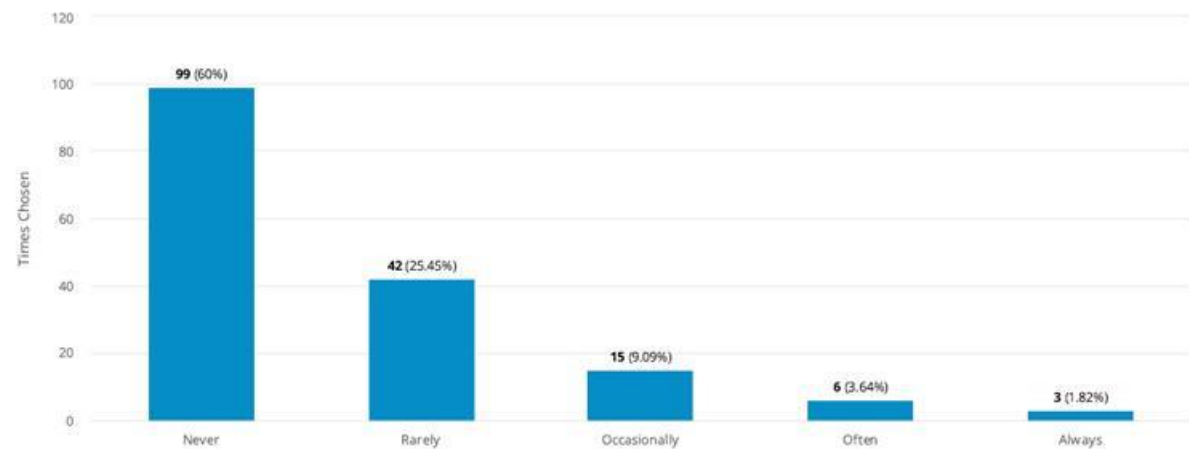

Supplement: Supplementary file 1 [file Datasheet1.pdf]
